# Role of PsrA in physiology and virulence regulation in *Pseudomonas aeruginosa* and other bacterial species

**DOI:** 10.1128/jb.00470-25

**Published:** 2026-03-11

**Authors:** Selene García-Reyes, Miguel Cocotl-Yáñez, Rodolfo García-Contreras, Israel Castillo-Juárez, Bertha González-Pedrajo

**Affiliations:** 1 Departamento de Genética Molecular, Instituto de Fisiología Celular, Universidad Nacional Autónoma de México, Ciudad Universitaria, Ciudad de México, Mexico; 2 Departamento de Microbiología y Parasitología, Facultad de Medicina, Universidad Nacional Autónoma de México, Ciudad Universitaria, Ciudad de México, Mexico; 3 Programa de Investigadoras e Investigadores por México Secihti, Instituto de Ciencias Básicas e Ingeniería, Universidad Autónoma del Estado de Hidalgo, Mineral de la Reforma, Hidalgo, Mexico; Dartmouth College Geisel School of Medicine, Hanover, New Hampshire, USA

**Keywords:** *Pseudomonas*, PsrA, virulence, pathogenesis, secretion

## Abstract

*Pseudomonas aeruginosa* is an opportunistic pathogen with a complex regulatory network controlling its physiology and virulence. A key component of this network is PsrA, a TetR-family transcriptional regulator initially identified as an activator of *rpoS*. PsrA participates in multiple processes, including stress responses, fatty acid metabolism, quorum sensing, and secretion-mediated mechanisms such as the type III secretion system (T3SS). Depending on promoter context and environmental conditions, PsrA functions as both an activator and a repressor, integrating metabolic cues, particularly long-chain fatty acids, to modulate gene expression. In *P. aeruginosa*, PsrA indirectly influences quorum sensing through acyl-CoA accumulation and directly represses *lasR* and *rhlR*, while also activating the *exsCEBA* operon, required for T3SS expression during acute infection. PsrA further influences *rsmZ* expression, modulating RsmA activity and affecting pyocyanin production and biofilm-associated traits and has been linked to integrase expression in integrons. Beyond *P. aeruginosa*, PsrA orthologs and PsrA-like regulators in genera such as *Azotobacter*, *Legionella*, *Xanthomonas*, and *Stenotrophomonas* display both conserved and divergent regulatory roles, particularly in lipid metabolism and stress adaptation. These findings support PsrA as a context-dependent regulatory integrator in well-characterized T3SS-positive *P. aeruginosa* lineages, underscoring important gaps in our understanding of its regulation, evolutionary diversification, and potential relevance for future anti-virulence approaches, although direct therapeutic exploitation remains to be validated.

## INTRODUCTION

*Pseudomonas aeruginosa* is a gram-negative bacterium known for causing severe infections due to its remarkable metabolic versatility and large genome (~6.3 Mb). *P. aeruginosa* synthesizes and utilizes multiple virulence traits that enable it to evade the host immune response, establish infections, and compete with other microorganisms for nutrients. Given the significant role of virulence factors in the establishment of infections, the phylogenetic profile of *P. aeruginosa* is relevant to its pathogenic potential. This genetic heterogeneity underlies the remarkable pathogenic versatility of *P. aeruginosa* populations. Based on its core genome, *P. aeruginosa* has been proposed to comprise five major clades, with clade 1 represented by the PAO1 reference strain, clade 2 by PA14, clade 3 by PA7, and clade 5 by PA39. However, the existence and definition of clade 4 remain uncertain (1, 2).

This clinical relevance is underscored by the inclusion of *P. aeruginosa* in the World Health Organization (WHO) 2024 list of high-priority pathogens, which highlights bacteria for which the development of new antibiotics is urgently needed (3). To support its pathogenic lifestyle, *P. aeruginosa* regulates virulence through multiple molecular mechanisms, including quorum sensing (4, 5), alternative sigma factors, transcription factors (6–9), the type III secretion system (T3SS), the type VI secretion system (10–12), two-component systems, such as GacS/GacA (10), and one-component systems, in which a single cytosolic protein integrates both sensing and regulatory functions. Among these, the transcriptional regulator PsrA plays a significant role. Discovered as a positive regulator of *rpoS* expression in *Pseudomonas putida* WCS358, PsrA (Pseudomonas Sigma Regulator) has been studied for its contribution to the physiology and virulence of *Pseudomonas* species. Despite numerous studies since its discovery, a comprehensive review of PsrA’s roles in *P. aeruginosa* virulence regulation remains lacking.

By linking lipid-derived metabolic cues to quorum sensing, sigma factor activity, and secretion system regulation, PsrA exemplifies how one-component regulators enable flexible lifestyle transitions in *Pseudomonas* species. Consistent with this integrative role, PsrA controls fatty acid β-oxidation, modulates quorum-sensing hierarchies, regulates secretion systems associated with acute infection, and influences integron-mediated resistance. Together, these regulatory activities link metabolic state, virulence-associated pathways, and stress adaptation at multiple levels of gene control. This convergence of regulatory functions positions PsrA as a key node within the global regulatory network of T3SS-positive *P. aeruginosa* strains and underscores the need for a focused synthesis of its roles and regulatory mechanisms.

This review aims to compile and analyze the roles of PsrA in the opportunistic pathogen *P. aeruginosa* and other bacterial species, with a particular focus on its control of virulence, quorum sensing, and its involvement in the regulation of genes associated with antibiotic resistance.

## PsrA INTEGRATES FATTY-ACID AVAILABILITY AND ENVIRONMENTAL STRESS INTO GLOBAL REGULATORY NETWORKS IN *PSEUDOMONAS*

PsrA is a 25.7 kDa protein (UniProtKB accession: Q9HZJ9) belonging to the TetR-family of transcription factors. This family of proteins has a conserved helix-turn-helix DNA-binding motif at the N-terminus and a variable ligand-binding site at the C-terminal domain (13). The TetR-family of transcription factors serves as activators or repressors of gene expression (13). For example, in *P. putida*, PsrA acts as a repressor by binding to the palindromic sequence C/GAAACN_2–4_ GTTTG/C (PsrA binding site) located between the −18 and +20 sites in the *psrA* promoter. This binding prevents RNA polymerase from accessing the DNA sequence of the promoter region, thereby inhibiting transcription (7).

Structural predictions from AlphaFold (accession number: Q9HZJ9) indicate that PsrA is composed of two well-defined α-helical domains with high confidence. The N-terminal region adopts a fold classified by CATH as homeodomain-like, reflecting structural similarity to helix–turn–helix DNA-binding motifs, while the C-terminal region corresponds to a TetR-family-like regulatory domain involved in dimerization and ligand binding. Importantly, the homeodomain-like annotation refers to structural architecture rather than functional homology to eukaryotic homeodomain transcription factors. This modular organization is consistent with the regulatory versatility of PsrA, although experimental validation of structure–function relationships is still lacking.

**Fig 1:**
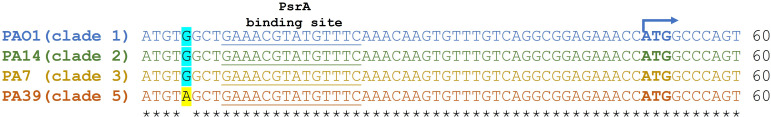
Conservation of the PsrA binding site across major *Pseudomonas aeruginosa* clades. Representative strains from clades 1 (PAO1), 2 (PA14), 3 (PA7), and 5 (PA39) were aligned. The conserved PsrA binding site is underlined, the psrA start codon (ATG) is shown in bold, and the G→A substitution characteristic of clade 5 is highlighted in yellow. Asterisks indicate fully conserved nucleotides.

In addition to *P. putida*, the presence of PsrA binding sites in the *psrA* promoter has also been reported in *P. aeruginosa* and *Pseudomonas chlororaphis* (7, 14). In *P. aeruginosa* PAO1, the PsrA binding site consists of the palindromic sequence GAAACN_4_GTTTC and is located at position −28 site relative to the ATG start codon of the *psrA* gene. This binding sequence is highly conserved in clades 1, 2, 3, and 5 of *P. aeruginosa* (Fig. 1). However, the expression and regulation of *psrA* in strains belonging to clades 3 and 5 have not been studied.

The G to A substitution in the −46 site (four nucleotides upstream of the PsrA binding site, relative to the *psrA* start codon) is conserved in all the strains belonging to clade 5 reported in the Pseudomonas Genome Database (https://www.pseudomonas.com), except for *P. aeruginosa* CEC124, which belongs to clade 5 but retains the G in that position. This nucleotide change, found in most clade 5 strains, appears to be unique to this clade and is unlikely to affect regulation at the PsrA binding site. However, as mentioned earlier, it is necessary to study the regulation of PsrA in these unexplored clades.

The TetR transcription factor has a ligand-binding site in the C-terminal domain, which, upon ligand binding, undergoes a conformational change altering its regulatory activity (13). As a member of the TetR family, PsrA interacts with oleic acid at its ligand-binding site, leading to dissociation of PsrA from the promoter of the *fadBA5*, which encodes proteins involved in fatty acid β-oxidation, thereby lifting the transcriptional repression and reflecting its function as a one-component regulatory system (15). The regulation of the *psrA* gene has been minimally investigated. It is known that the two-component GacS/A system plays a role in *psrA* expression, at least in *P. chlororaphis*, where expression was abolished in a *gacS* mutant. This suggests a positive regulation by this two-component system, although it remains unclear whether the regulation occurs directly or indirectly (14). In contrast, no differences in translational activity of a P*psrA::lacZ* fusion were observed between a *gacA* mutant and a wild-type strain of *Pseudomonas fluorescens* CHA0, indicating that GacS/A does not regulate *psrA* expression in *P. fluorescens* (16). On the other side, the effect of *gacS* or *gacA* mutants on *psrA* expression in *P. aeruginosa* remains unstudied.

A transcriptomic analysis conducted by Gicquel et al. (17) demonstrated that the sigma factor SigX, which is involved in stress responses such as membrane remodeling during biofilm production and in the expression of fatty acid biosynthetic genes, also positively regulates *psrA* expression. Bouffartigues et al. (18) reported that under cold conditions, the levels of *sigX* mRNA increase, while *psrA* mRNA levels decrease. This suggests a transcriptional derepression of the β-oxidation pathway.

Under cold stress, SigX regulates the expression of genes involved in lipid synthesis for membrane preservation and utilizes them as an energy source by derepressing fatty acid β-oxidation, potentially through a direct or indirect mechanism that reduces *psrA* mRNA. This suggests a complex regulatory effect of SigX on *psrA*, warranting further investigation into how factors like temperature and stress influence PsrA’s regulatory role in *Pseudomonas* species (18).

Beyond its direct regulatory targets, PsrA is embedded within the broader transcriptional architecture of *P. aeruginosa*. Large-scale transcriptomic analyses combining RNA-seq of multiple transcription factor mutants have revealed that *psrA* is differentially expressed in mutants of several global regulators, including ExsA, GbdR, LasR, PchR, and PhoB (19). Co-occurrence analysis demonstrated that the overlap of *psrA* differential expression across these mutants is statistically significant, indicating coordinated regulatory input.

These regulators represent distinct yet interconnected physiological modules: ExsA positively controls the T3SS (20), LasR is part of the quorum-sensing networks (21), PchR regulates iron acquisition through pyochelin (22), GbdR directly controls choline acquisition from host phospholipids (23), and PhoB mediates responses to phosphate limitation (24). The convergence of these regulatory pathways on *psrA* expression supports the view that PsrA functions as an integrative node linking environmental sensing, metabolic status, and virulence-associated gene expression. While the RNA-seq-based co-regulation does not imply direct promoter binding, it places PsrA within a highly interconnected regulatory network that coordinates adaptive responses during infection and environmental stress (19). The intricate regulation of PsrA by various environmental and genetic factors highlights its significant role in the adaptive and pathogenic responses of *Pseudomonas* species. To fully understand the extent of PsrA’s influence, it is essential to examine its regulon, that is., the collection of genes directly or indirectly regulated by PsrA. In the following section, we will explore the PsrA regulon, detailing the specific genes and pathways it controls and their contributions to the physiological and virulent characteristics of *P. aeruginosa* and other related bacterial species.

Thus, *psrA* expression is finely tuned by metabolic and stress-related signals, allowing PsrA to function as a one-component sensor that integrates fatty-acid availability and membrane stress into adaptive transcriptional responses.

## PsrA REGULON AS A METABOLIC INTEGRATION COORDINATING STRESS ADAPTATION, SOCIAL BEHAVIOR, VIRULENCE, AND RESISTANCE

### PsrA integrates fatty-acid metabolism with the RpoS–Gac/Rsm stress regulatory axis

The alternative sigma factor RpoS (σ^38^) plays a crucial role during the stationary growth phase and under stress conditions. It competes with the core RNA polymerase and activates genes associated with the general stress response and virulence. In *P. aeruginosa*, RpoS negatively regulates the production of pyocyanin by repressing the *pqsABCDE* operon and biofilm, which are vital virulence factors contributing to pathogenicity and antibiotic resistance (25–27).

The first evidence of PsrA acting as a positive regulator of *rpoS* was observed in *P. putida* WCS358 and *P. aeruginosa* PAO1, where *psrA* mutants showed a 90% reduction in *rpoS* transcription (8, 28). Furthermore, Western blot analysis confirmed that the RpoS protein was not detected in a *P. chlororaphis psrA* mutant, highlighting the positive regulation of *rpoS* by PsrA in different *Pseudomonas* species (29).

Subsequently, the PsrA binding site on the *rpoS* promoter was determined using a DNase I footprinting assay in *P. putida*, revealing a palindromic sequence (TTCAAACN_4_GTTTGAA) located between positions −59 and −35 relative to the *rpoS* ATG start codon. Electrophoretic mobility shift assays (EMSAs) with purified His6-PsrA confirmed its binding as a homodimer to the *rpoS* promoter (7).

**Fig 2:**
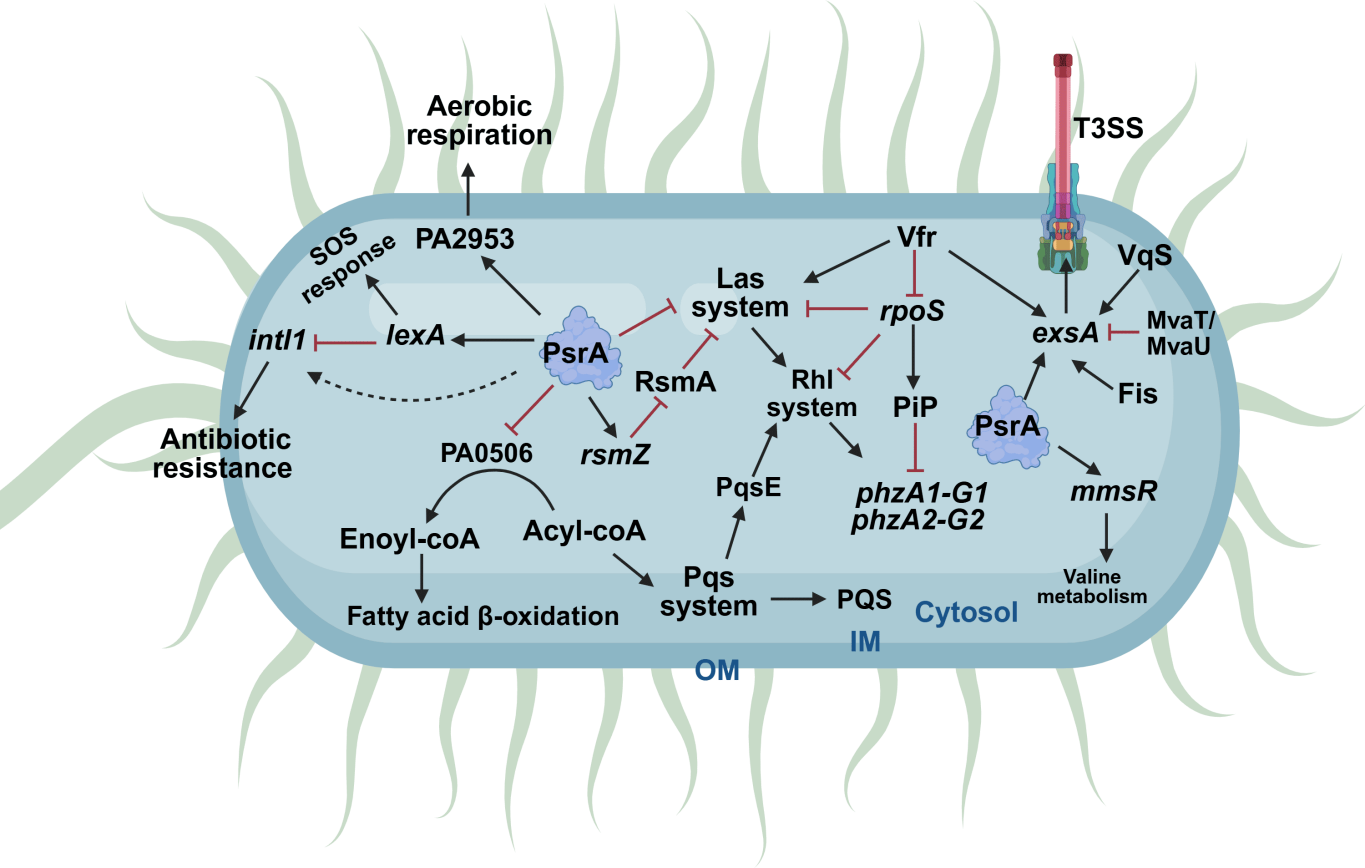
Integrated regulatory network controlled by PsrA in *P. aeruginosa*. The schematic summarizes how PsrA connects fatty acid metabolism with global regulatory circuits controlling quorum sensing, stress adaptation, and acute virulence. PsrA directly or indirectly regulates genes involved in fatty acid β-oxidation, leading to acyl-CoA accumulation and modulation of the Las, Rhl, and Pqs quorum-sensing systems, including PqsE-dependent activation of RhlR. Through regulation of rpoS and interaction with the Gac/Rsm pathway via rsmZ and RsmA, PsrA fine-tunes stress responses, secondary metabolism, and virulence-associated traits such as pyocyanin production. PsrA also influences type III secretion system expression through activation of the exsCEBA operon and integrates additional inputs from regulators such as Vfr, Vqs, MvaT/MvaU, and Fis. Arrows indicate positive regulation, red blunt-ended lines indicate repression, and dashed lines denote indirect regulatory effects. OM, outer membrane; IM, inner membrane. Created in BioRender.

In addition to PsrA, other proteins have been reported to regulate *rpoS* in *P. aeruginosa* (Fig. 2). For instance, Vfr acts as a negative regulator of *rpoS* transcription during the early exponential phase (28). In addition, in *P. aeruginosa* PAO1, the GacS/A system has been reported to regulate *rpoS* transcription through quorum sensing, because the *lasR* gene, which encodes the LasR transcription factor, is not expressed in a *gacA* mutant (30), and *rpoS* is not expressed in a *lasR* mutant (31). However, in 2003, Bertani et al. (28) also reported that in *P. aeruginosa,* a *gacA* mutant showed no changes in *rpoS* expression, suggesting that this system does not regulate *rpoS*.

Conversely, it has been reported that the GacS/A system regulates the expression of *rpoS* in *P. fluorescens* Pf-5 (32). Interestingly, in the *P. fluorescens* CHA0 *gacA* mutant, the expression of *psrA* was not affected (16), implying that the GacS/A regulation of *rpoS* in this strain is not through PsrA.

Through its control of *rpoS*, PsrA couples metabolic status to global stress adaptation, enabling coordinated transcriptional reprogramming during nutrient limitation and stationary-phase-like conditions.

Beyond its direct transcriptional activation of *rpoS*, PsrA is also embedded within the Gac/Rsm post-transcriptional regulatory network. This central signaling axis fine-tunes RpoS activity and controls social behaviors, secondary metabolism, and virulence in *Pseudomonas* species. Through this multilayered regulation, metabolic cues sensed by PsrA are translated into coordinated population-level responses during stress and stationary-phase-like conditions.

In *P. aeruginosa*, the two-component system GacS/A plays an important role in cell communication and secondary metabolite production. GacS is a histidine kinase protein that receives a not-yet-characterized signal and phosphorylates the response regulator GacA (33). Phosphorylated GacA can activate the transcription of two non-coding small RNAs, *rsmY* and *rsmZ*, which bind to the RNA-binding proteins RsmA and RsmN. The Gac/Rsm signaling cascade controls its targets at the post-transcriptional level and acts as a lifestyle modulator (34).

In *P. aeruginosa* PAO1, RsmA controls pyocyanin synthesis by negatively regulating the expression of the Las and Rhl systems (Fig. 2) (35). In addition, in the marine strain ID4365, RsmA regulates pyocyanin production by negatively regulating *phzM* and *phzS* expression (36), which are involved in pyocyanin production from phenazine-1-carboxylic acid (37). Interestingly, in this environmental strain, the translation of RpoS is positively regulated by RsmA (Fig. 2) (36). In *P. fluorescens* CHA0, it has been reported that, in addition to GacA, PsrA regulates the expression of *rsmZ*. This regulation occurs directly since PsrA binds to the promoter region of *rsmZ* (Fig. 2) (16).

While the regulation of PsrA on *rsmZ* has been reported in *P. fluorescens* CHA0, it is evident that PsrA can control *rpoS* expression through two distinct mechanisms. First, PsrA acts as a transcriptional activator of *rpoS* expression, and second, PsrA seems to negatively regulate *rpoS* by activating transcription of *rsmZ*, which in turn binds to RsmA to positively regulate translation of *rpoS* mRNA (Fig. 2) (36).

Together, these observations place PsrA as a key regulator that interfaces with the Gac/Rsm signaling cascade to fine-tune RpoS levels and quorum-dependent behaviors, reinforcing its role as a central integrator of metabolism, stress adaptation, and social coordination in pseudomonads.

### Metabolic control of quorum sensing by PsrA

In *P. aeruginosa*, virulence factor genes are tightly regulated by the Las, Rhl, and Pqs quorum-sensing systems. Among them, the Las system takes the lead in the signaling cascade, particularly in nutrient-rich environments (5, 21, 38, 39). Under phosphate-limiting conditions, the Las system becomes dispensable, and the Rhl system assumes a dominant position at the top of the regulatory cascade (40).

The Las system consists of the LasR and LasI proteins. LasR is a transcriptional regulator, while LasI is a synthase that produces the autoinducer N(3-oxododecanoyl) homoserine lactone (3-oxo-C12-HSL). LasR, when bound to 3-oxo-C12-HSL, is converted to its active form to regulate the transcription of its target genes (41). One of these targets is the Rhl system, composed of the RhlR transcriptional regulator and the RhlI synthase, which synthesizes the N-butyryl-homoserine-lactone (C4-HSL) autoinducer. RhlR is active as a regulator when in a complex with its autoinducer C4-HSL (42, 43). This system regulates pyocyanin production through the expression of the *phzA1B1C1D1E1F1G1* (*phzA1*) and *phzA2B2C2D2E2F2G2* (*phzA2*) operons (37, 44), which convert the chorismic acid into phenazine-1-carboxylic acid. Next, phenazine-1-carboxylic acid is converted into 5-methylphenazine-1-carboxylic acid betaine by the PhzM enzyme and finally transformed into pyocyanin via the PhzS enzyme (37).

Finally, the Pqs system is the last to be activated, and unlike the previous two systems, it synthesizes and responds to a quinolone-type autoinducer known as Pseudomonas quinolone signal (PQS), as well as its precursor molecule 2-heptyl-4-quinolone (HHQ). HHQ is synthesized by the enzymes encoded in the *pqsABCDE* operon and converted to PQS by the PqsH monooxygenase encoded by the *pqsH* gene (45–47). The LysR-type protein PqsR is the transcriptional regulator of the Pqs system. The three quorum-sensing systems are interconnected; the Las system positively regulates the Pqs and Rhl systems, while the Rhl system regulates the Pqs system in a negative form, and in turn, the Pqs system has a positive effect on RhlR through the PqsE protein encoded in the *pqsABCDE* operon, which increases its regulatory activity of RhlR (4, 48–50). Moreover, quorum sensing is positively regulated by the transcriptional factor Vfr, which regulates *lasR* independently of cAMP (51) and the expression of *rhlR* (52).

It has been reported that PsrA indirectly regulates quorum sensing in *P. aeruginosa* in different ways (Fig. 2). First, by repressing the expression of the PA0506 gene, which encodes an acyl-CoA dehydrogenase, this enzyme converts the acyl-CoA moieties into enoyl-CoA in the first step of fatty acid β-oxidation. This enzymatic activity results in the accumulation of acyl-CoA, which is the precursor of the PQS autoinducer (53). This effect was demonstrated in stationary-phase cultures grown in rich media, where metabolic flux through fatty acid catabolism is prominent, and PQS production is maximal. Under these conditions, supplementation with long-chain fatty acids or deletion of PA0506 restores PQS production in a *psrA* mutant, supporting an indirect, metabolism-dependent role of PsrA in PQS regulation. In relation to pathogenicity, this is important because the presence of PQS has been shown to contribute to the production of virulence factors in *P. aeruginosa* (45).

Second, PsrA directly represses quorum sensing by binding to the *lasR* promoter, thereby reducing *lasR* transcription. A transcriptomic analysis of *P. aeruginosa* PAO1 overexpressing *psrA* revealed downregulation of the master quorum-sensing regulators *lasR* and *rhlR*, accompanied by reduced elastase, pyocyanin, and swarming activity. Furthermore, using an EMSA experiment, it was demonstrated that PsrA directly binds to the *lasR* promoter, while the addition of oleic acid inhibits this binding and restores quorum-sensing-related phenotypes to wild-type levels. These findings highlight that PsrA negatively regulates *lasR* expression, with oleic acid acting as a key signaling molecule that modulates this regulatory pathway (54). These experiments were conducted under conditions where the Las quorum-sensing system is active, corresponding to exponential to early stationary growth phases, in which classical AHL-mediated signaling predominates. Consistently, previous reports indicated that PsrA binding to oleic acid increases C4-HSL levels in PAO1 (55), which is now understood to result from the relief of PsrA-mediated repression of *lasR*.

In addition to these mechanisms, PsrA also influences quorum sensing through RpoS, a sigma factor that affects the expression of the *phz1* and *phz2* operons. In a *rpoS* mutant, *phz1* expression and phenazine production were elevated due to increased *lasR* translation, suggesting that RpoS has a negative effect on quorum-sensing regulation (25). In contrast, it was demonstrated that RpoS positively regulates pyocyanin production from the *phzA2* operon through the Pip protein (56).

Furthermore, overexpression of *psrA* in PAO1 was associated with pyocyanin overproduction (54). Initially, this effect was attributed to a possible positive regulatory role of RpoS on quorum sensing, because PsrA acts as a positive regulator of *rpoS* (8, 28); however, it is important to note that RpoS overexpression has been linked to a decrease in pyocyanin levels (27). A more plausible explanation is that *psrA* overexpression may promote the activation of the small RNA RsmZ, which sequesters RsmA and thereby promotes pyocyanin production. Notably, PsrA has been reported to directly regulate *rsmZ* expression in other *Pseudomonas* species (16), suggesting that a similar mechanism operates in *P. aeruginosa* (see “PsrA integrates fatty-acid metabolism with the RpoS–Gac/Rsm stress regulatory axis,” above, for details). In addition, recent findings reported that in a *rpoS* mutant of *P. aeruginosa* PAO1, PqsE levels increase, leading to stabilization of RhlR and enhanced transcription of the *phz1* operon under low phosphate medium (PPGAS medium) (27, 29). These observations highlight the complexity of the regulatory interplay between PsrA, RpoS, RsmZ, and PqsE in modulating pyocyanin biosynthesis in *P. aeruginosa*.

Interestingly, in *P. chlororaphis*, PsrA regulates quorum sensing through RpoS activity. Both *psrA* and *rpoS* mutants fail to produce the quorum-sensing autoinducer N-hexanoyl-L-homoserine lactone (C6-HSL), whereas overexpression of *rpoS* in the *psrA* mutant restores its production, indicating a positive role of RpoS in *P. chlororaphis* quorum-sensing regulation (29).

Overall, the indirect regulation of quorum sensing via PQS, the direct repression of *lasR*, and the modulation through RpoS underscore the multifaceted role of PsrA in coordinating virulence-associated pathways in *P. aeruginosa* T3SS-positive strains. Thus, PsrA does not exert a uniform effect on quorum sensing but rather rewires quorum-sensing outputs according to metabolic state, growth phase, and the prevailing virulence mechanism. By modulating quorum-sensing hierarchies in response to fatty-acid-derived metabolic cues, PsrA links cellular metabolic state to population-level social behaviors and multicellular coordination.

### PsrA-dependent control of secretion systems during acute infection

The T3SS is a primary virulence determinant during acute infection of *P. aeruginosa,* which translocates cytotoxic proteins, termed effectors, across the host cell membrane (12, 34). In *P. aeruginosa*, the T3SS regulon consists of 40 genes that encode the secretion and translocation machinery, regulatory factors, effectors, and chaperones (57). These genes are organized into five transcriptional units, and each is under the direct transcriptional control of the transcription factor ExsA, which is encoded in the *exsCEBA* operon (58, 59). The ExsA-dependent expression of T3SS genes is induced under low calcium conditions or upon contact of *P. aeruginosa* with its host cells (58). Because T3SS expression is energetically costly and tightly linked to acute virulence, its regulation provides a key entry point to understand how metabolic regulators such as PsrA influence infection strategies.

Transcription of *exsA* occurs from the promoter of the operon *exsCEBA* (P*exsC*), where ExsA positively regulates its transcription (57, 59, 60). Using a mobility shift assay, it was shown that PsrA positively regulates *exsA* expression by binding to P*exsC* (Fig. 2) (61). Additionally, there is an internal promoter upstream of *exsA* that is positively regulated by Vfr (62), Fis, and Vqs and negatively by MvaT/MvaU (59, 63).

From a medical standpoint, studying the regulation of PsrA on the T3SS is critically important due to its role as a key virulence factor in *P. aeruginosa* during acute infections (12, 64, 65). An *exsA* mutant completely suppresses the transcription of T3SS genes and exhibits a significant reduction in T3SS-dependent cytotoxicity in eukaryotic cells (66). This observation holds for *P. aeruginosa* strains belonging to clades 1 and 2. Conversely, clades 3 and 5 strains possess the type V secretion system (T5bSS) ExlB/A instead of the T3SS, where the ExlA toxin is secreted (67).

This two-partner secretion system likely compensates for the cytotoxic function of T3SS in *P. aeruginosa*. Interestingly, the secretion of the ExlA toxin is positively regulated by Vfr/cAMP (68), like the regulation of T3SS. Nevertheless, it is established that both systems are mutually exclusive, as no reports have indicated their coexistence in the same strain (1). The apparent mutual exclusivity between T3SS and the ExlA two-partner secretion system in *P. aeruginosa* raises evolutionary and functional questions. One possible explanation is that maintaining both systems would impose a significant metabolic burden, as both secretion systems require biosynthetic investment and regulatory control. Alternatively, because both systems are anchored to the membrane, their simultaneous presence could compromise membrane integrity and lead to cellular damage. In addition, both systems serve a similar function, host cell intoxication, suggesting functional redundancy. A second possibility is that the presence of the ExlA two-partner secretion system reflects a distinct mode of host interaction rather than adaptation to a specific ecological niche. However, ExlA-positive *P. aeruginosa* strains have been isolated from a wide range of sources, including human infections, healthy animals, and environmental reservoirs, indicating that ExlA is not restricted to non-clinical or environmental settings (69, 70).

Functionally, ExlA-mediated cytotoxicity relies on pore formation and epithelial barrier disruption (71, 72), which contrasts with the effector-mediated intracellular targeting characteristic of T3SS (59, 73). These mechanistic differences suggest that the T3SS and ExlA may represent alternative strategies to achieve host damage and dissemination, each optimized for different host cell types, stages of infection, or immune contexts.

Finally, regulatory incompatibility may also contribute to this exclusivity. Both the T3SS and ExlA expression are controlled by the cAMP–Vfr regulatory network, and cross-regulation or interference at this level may prevent the stable coexistence of both systems within the same strain. Experimental testing of these hypotheses will be necessary to determine the evolutionary pressures shaping secretion system distribution in *P. aeruginosa*.

Furthermore, it remains unknown whether PsrA is involved in the regulation of the ExlB-ExlA two-partner secretion system. This gap precludes extrapolating the central regulatory role of PsrA observed in T3SS-positive strains to ExlA-positive clades.

To gain comprehensive insights into the virulence of *P. aeruginosa* during acute infections, it is crucial to conduct more studies about PsrA in T3SS regulation. The existence of an alternative two-partner secretion system, alongside its exclusive relationship with T3SS, necessitates in-depth exploration, including the potential role of PsrA in regulating this system. PsrA-dependent regulation of secretion systems illustrates how metabolic information is integrated into virulence programs, allowing *P. aeruginosa* to align energy availability with aggressive infection strategies.

### PsrA-mediated control of fatty acid β-oxidation links metabolism to virulence and energy homeostasis

As mentioned previously, PsrA indirectly regulates the stationary phase by influencing RpoS transcription, which in turn controls several aspects of bacterial activity. During this period, bacteria encounter challenges in obtaining carbon sources necessary for energy production. Consequently, the bacteria modulate genes responsible for acquiring carbon sources and synthesizing precursors essential for cell biosynthesis. Among the mechanisms involved, fatty acid β-oxidation plays a crucial role in producing intermediates and electron donors required for energy generation. This process allows the bacteria to meet their energy demands even in the presence of limited carbon sources (74). In *P. aeruginosa*, this metabolic pathway is tightly regulated by PsrA, positioning fatty acid β-oxidation as a central hub linking nutrient availability to stress adaptation and virulence.

Regarding this, Kojic et al. (75) reported that PsrA directly regulates fatty acid β-oxidation, controlling the expression of *PA0506* (probable acyl-CoA dehydrogenase), *PA2952–PA2951* operon (encoding an electron transfer flavoprotein β-subunit and α-subunit, respectively), *PA2953* (encoding an electron transfer flavoprotein–ubiquinone oxidoreductase), and *PA3571* (encoding the transcriptional regulator MmsR) (Fig. 2). The *PA0506* gene and the *PA2952–PA2951* operon are negatively regulated, whereas *PA2953* and *mmsR* promoters are positively controlled by PsrA (75). This metabolic regulation acquires relevance during lung infection, where host-derived fatty acids constitute a major and readily accessible nutrient source for *P. aeruginosa* (76, 77).

In the context of lung infection and host-pathogen interactions, pulmonary surfactants play a vital role in lung function and comprise approximately ~10% surfactant proteins and ~90% lipids (77). They serve as a significant carbon and nitrogen source for *P. aeruginosa* during colonization of the lungs of cystic fibrosis patients (77, 78). Use of these pulmonary surfactants by *P. aeruginosa* modifies the virulent behavior of the bacterium by indirectly regulating its quorum-sensing systems (77).

Juarez-Rodriguez et al. (79) proposed that during the late logarithmic phase in *P. aeruginosa* PA14, PsrA represses fatty acid β-oxidation by downregulating the expression of *PA0506*, which encodes an acyl-CoA dehydrogenase responsible for converting acyl-CoA molecules into enoyl-CoA during the initial stage of fatty acid β-oxidation. This repression leads to the accumulation of acyl-CoA, a precursor of the PQS autoinducer, thereby promoting PQS production and triggering PQS-dependent virulence factor production (Fig. 2) (53). Furthermore, during this phase, PsrA exerts a positive regulatory influence on the *exsCEBA* operon, promoting the transcription of T3SS genes and facilitating the secretion of ExoU. This lipase can elevate the concentration of lauric and myristic acids from the host cell lipids in the microenvironment (79).

Subsequently, in the stationary phase, high concentrations of lauric and myristic acids released by host cells are observed (79). These fatty acids have the potential to bind to PsrA, forming a complex that subsequently reduces its transcriptional activity (15), releasing the repression of fatty acid degradation and allowing these fatty acids to be used as an energy source.

Together, these findings support a model in which PsrA functions as a metabolic switch, transiently repressing fatty acid β-oxidation to favor acute virulence and later permitting lipid catabolism to sustain energy production and long-term survival.

### PsrA-mediated integration of metabolic and stress signals impacts integron regulation and antibiotic resistance

Antibiotic resistance in *P. aeruginosa* is tightly linked to its ability to sense and respond to environmental stress while maintaining metabolic flexibility and using mobile elements such as transposons, plasmids, and integrons for the acquisition of resistance genes (80). Beyond its established role in virulence regulation, PsrA has emerged as a regulatory node that connects metabolic state with stress-response pathways, influencing genome plasticity and long-term persistence. In this context, integrons represent a key adaptive mechanism, enabling rapid acquisition and rearrangement of resistance determinants under selective pressure. Evidence from *Pseudomonas* species indicates that PsrA impacts integron activity through modulation of stress-associated regulators, positioning this transcription factor at the intersection of metabolism, stress adaptation, and antimicrobial resistance.

The resistance acquired by integrons is mediated by site-specific recombination carried out by the integrase IntI, a tyrosine recombinase. The integrase (encoded by the *intI1* gene) is a crucial enzyme involved in the recombination process between different DNA substrates. Intl facilitates recombination between double-stranded (*attI*) and single-stranded (*attC*) substrates. In addition to this primary function, Intl can process recombination events between two double-stranded substrates or two single-stranded substrates (81). This versatility in substrate recognition and processing underscores the important role of integrase in mediating genetic rearrangements and promoting genetic diversity.

Integrase transcription is repressed by the transcription factor LexA (81). In *P. aeruginosa,* the *lexA* gene is encoded in the opposite direction of *psrA,* sharing the same intergenic region. In the context of *P. putida* WCS358, a study conducted by Novovic et al. (82) revealed that PsrA exerts a positive influence on both the *intI1* and *lexA* promoters during the stationary phase. This suggests a potential indirect regulatory mechanism in which PsrA impacts *intI1* expression by modulating *lexA* expression (Fig. 2), likely in conjunction with other regulatory factors. Although PsrA positively regulates LexA, which is the negative regulator of *intI1* integrase expression, the overall effect of PsrA on integrase transcription is positive (Fig. 2).

These findings shed light on the complex interplay between different regulatory proteins and highlight the intricate control mechanisms governing integrase gene expression in *P. putida* WCS358, which may differ in their regulation and organization from those present in *P. aeruginosa*.

In addition, LexA is involved in the SOS response, a mechanism to repair DNA when it is damaged by different types of stress (83). Therefore, PsrA may play a pivotal role in the survival of certain bacteria. Further investigations are warranted to elucidate the precise molecular interactions underlying these regulatory mechanisms. Together, these observations extend the role of PsrA beyond acute virulence regulation, highlighting its contribution to stress-induced genome plasticity and antimicrobial resistance. By integrating metabolic cues with SOS and integron-associated pathways, PsrA may facilitate adaptive strategies that promote persistence and survival in fluctuating and hostile environments.

## ROLE OF PsrA IN BACTERIAL SYSTEMS BEYOND *PSEUDOMONAS AERUGINOSA*

### Azotobacter vinelandii

*A. vinelandii* belongs to the Pseudomonadaceae family. This bacterium produces two important polymers: alginate and poly-β-hydroxybutyrate (PHB) (84). Additionally, this bacterium can undergo an encystment process that enables it to survive under less favorable conditions, such as desiccation and nutrient deprivation. In the cyst, alginate forms two protective layers around the bacterial cell, and the poly-β-hydroxybutyrate is stored as a carbon resource. In addition, the phenolic lipids alkylresorcinols are synthesized and replace the phospholipids of the membrane (85). Alginate, PHB, and alkylresorcinols synthesis is tightly controlled by different global regulators and the alternative sigma factor RpoS. It was reported that *rpoS* inactivation decreases PHB accumulation and alkylresorcinols production and also abolishes the encystment process (86–88). In addition, RpoS drives the expression of *algD*, the key gene in alginate biosynthesis, since one of the three *algD* promoters is *rpoS* dependent. Furthermore, the genes that code for the seven alginate-epimerases, which convert mannuronic acid into guluronic acid, are under *rpoS* control (89, 90). Thus, RpoS plays an important role in the physiology of this bacterium.

Since *A. vinelandii* is closely related to that of *Pseudomonas* spp., it is not surprising that the *A. vinelandii* genome contains a gene that codes for a protein (WP_376942261.1) that shares 70% of amino acid identity and 80% similarity to PsrA from *P. aeruginosa* according to a BlastP analysis. This level of identity is comparable to, or even higher than, the average identity observed among different *Pseudomonas* species*,* which is 66% for *P. putida,* 49% for *P. fluorescens,* and 65% for *Pseudomonas syringae* (86, 91). Like *Pseudomonas* spp., PsrA in *A. vinelandii* is an activator of *rpoS* expression. Moreover, PsrA binds the *rpoS* promoter at a sequence resembling that found in *Pseudomonas* spp. In addition, negative PsrA autoregulation has also been described in *A. vinelandii* since PsrA specifically binds to the *psrA–lexA* intergenic region and represses *psrA* transcription; therefore, *psrA* inactivation relieves this repression and results in increased *psrA* expression. In addition, another regulator could be included, that is., *psrA* is regulated by GacS/A in *P. chlororaphis* (14), or SigX in *Pseudomonas* species (18). With regard to the phenotype, *psrA* inactivation only reduces 50% of alkylresorcinols production and is not able to abolish the encystment process. Moreover, the *rpoS* mutant strain is unable to survive in 300 mM hydrogen peroxide, but the *psrA* mutant can grow in this condition. The difference between these two mutant strains could be explained because *rpoS* possesses two promoters, one of them PsrA-dependent; therefore, in the *psrA* mutant strain, *rpoS* is still expressed from the second promoter, explaining the partial phenotypes (86).

**Fig 3:**
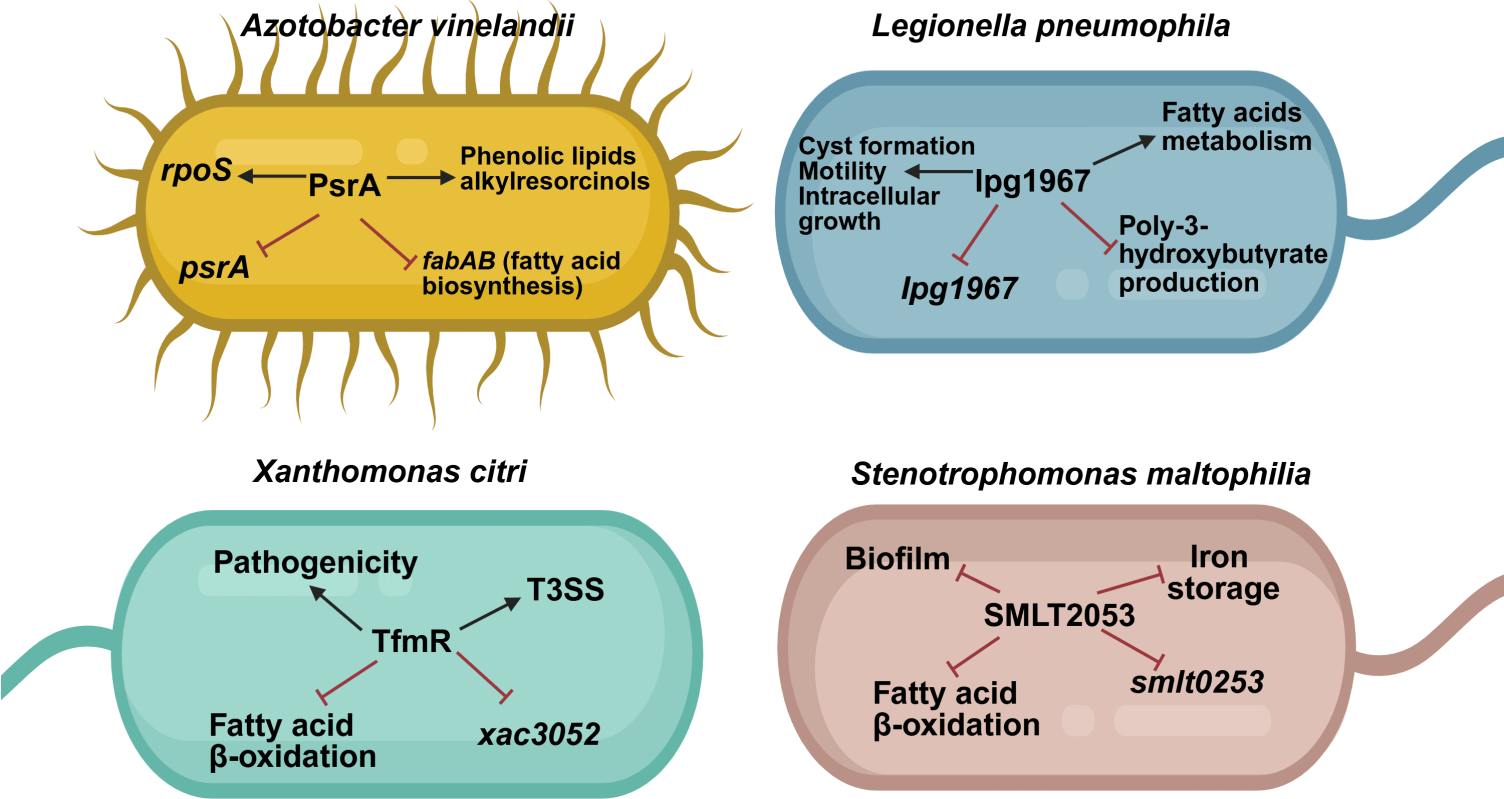
Conserved and divergent regulatory roles of PsrA homologs and PsrA-like TetR-family regulators in different bacterial species. The schematic summarizes key genes and biological processes controlled by PsrA or functionally related regulators in *Azotobacter vinelandii*, *Legionella pneumophila*, *Xanthomonas citri*, and *Stenotrophomonas maltophilia*. Across these organisms, PsrA homologs integrate fatty acid metabolism with diverse physiological outputs, including stress adaptation, intracellular growth, virulence, biofilm formation, iron storage, and secretion system regulation. The arrows indicate positive regulation, and red blunt-ended lines indicate repression, highlighting both conserved metabolic control and species-specific regulatory adaptations. Created in BioRender.

Finally, in *A. vinelandii,* PsrA negatively regulates the expression of the *fabAB* operon, which is involved in unsaturated fatty acid biosynthesis. When *psrA* is inactivated, it reduces its content. The growth is not affected, but this mutant shows a loss of cell viability during long-term growth. Control of PsrA on the *fabAB* operon is direct, as this protein can bind to the *fabA* promoter region (Fig. 3). Interestingly, PsrA in *A. vinlandii* does not affect the expression of β-oxidation genes as it does in *Pseudomonas* spp., highlighting the difference in the regulation by PsrA in these two bacterial genera (92).

### Legionella pneumophila

*L. pneumophila* is a facultative intracellular parasite of freshwater protozoa, infecting amoebae that serve as a niche for intracellular replication and protection from environmental conditions, including antibiotics, some chemicals, heat, and osmotic stress. In humans, *L. pneumophila* causes respiratory illness, especially in people who are immunocompromised or who have been exposed to contaminated water (93). The expression of genes associated with virulence and cyst morphogenesis is regulated by the sigma factor RpoS. *L. pneumophila* has a *psrA* ortholog named *Ipg1967*, and it is expressed as a monocistronic transcript that negatively regulates its expression, similar to *P. aeruginosa, P. putida* (7), and *A. vinelandii* (86). Curiously, the *L. pneumophila Ipg1967 (psrA*) promoter has two *Ipg1967* (PsrA) binding sites (94).

Another important aspect is that in *L. pneumophila*, PsrA is not involved in regulating *rpoS* expression. In this bacterium, *rpoS* has two promoters, but its complete regulation remains unknown, although RpoN has been implicated (94).

In addition, the PsrA binding site in target genes is different from the PsrA binding site in *Pseudomonas* (94). This phenomenon was also observed in *A. vinelandii,* suggesting a unique repertoire of target genes that evolved according to the needs of each species.

Graham et al. (94) reported that PsrA participates in the internalization of *L. pneumophila* (Fig. 3), since a *psrA* mutant affects the bacterial intracellular growth in *Acanthamoeba castellani* protozoa. The lack of *psrA* generates an increase in PHB inclusions in mature transmissive cysts (Fig. 3) (93) that are used as a carbon source (95). Moreover, PsrA positively regulates the bacterial proliferation of *L. pneumophila* in macrophages (83), the cyst morphogenesis, the quorum-sensing response, and fatty acid metabolism mediated by the acyl carrier protein Acpp2, which is involved in fatty acid biosynthesis.

### Xanthomonas citri

*X. citri* is a plant pathogen that mainly attacks citrus plants. To cause the infection, this bacterium employs the T3SS machinery to inject effector molecules into the host that produce erumpent corky necrotic lesions often surrounded by a chlorotic halo on the leaves, young stems, and fruits, which cause dark spots, defoliation, reduced photosynthetic rate, rupture of leaf epidermis, dieback, and premature fruit drop (96). The virulence related to the T3SS in *X. citri* is controlled by the master transcriptional regulator HrpG that controls the expression of HrpX, which subsequently controls the expression of T3SS genes and effectors (97). However, upstream of HrpG and HrpX, the T3SS is controlled by the TetR-family transcriptional factor TfmR (T3SS and Fatty acid Mechanism Regulator) (Fig. 3), encoded by the *xac3052* gene. Like PsrA of *P. aeruginosa*, TfmR can sense and bind to long-chain fatty acids, disrupting its transcriptional activity (98). Although TfmR and PsrA share low sequence identity (18%) and moderate similarity (32%), structural alignment of their AlphaFold-predicted models using TM-align revealed that both proteins adopt a conserved overall fold (TM-score = 0.57), consistent with their classification within the TetR family. This structural conservation, despite substantial sequence divergence, indicates that TfmR and PsrA are not close orthologs but rather functionally related regulators that have likely evolved convergently to link fatty acid metabolism with the control of virulence-associated genes. Disruption of *tfmR* resulted in reduced virulence in citrus plants that can be partially complemented by overexpression of *hrpG*. Through EMSA and qRT-PCR assays, it is known that TfmR indirectly regulates *hrpG* and *hrpX* (98).

In addition to T3SS, the TfmR negatively regulates the expression of *fadE, mhpC,* and *fadH* genes (Fig. 3), which are not involved in T3SS function but are involved in fatty acid catabolism. This repression is released when TfmR binds to long-chain fatty acids like oleic acid.

### Stenotrophomonas maltophilia

*S. maltophilia* is a highly prevalent environmental bacterium that has gained recognition as a significant nosocomial pathogen. It has been detected in various sources, including ventilatory equipment, nebulizers, and endoscopes (99).

The pathogenicity of *S. maltophilia* is attributed to its production of proteolytic enzymes, such as DNase, RNase, elastase, lipase, hyaluronidase, mucinase, and hemolysin. These enzymes potentially contribute to the severity of infections caused by this bacterium. Additionally, *S. maltophilia* exhibits a diffusible signal factor (DSF) system that responds to changes in cell density. This intricate system involves the synthesis and perception of diffusible signal molecules of the fatty acid type. It plays a pivotal role in regulating various virulence and adaptation behaviors in the bacterium (100). These factors play essential roles in the adherence to and invasion of epithelial cells, biofilm formation, and the development of antimicrobial resistance (99).

Overall, the multifaceted pathogenic mechanisms employed by *S. maltophilia* underscore its significance as an opportunistic pathogen, particularly in healthcare settings and among individuals with compromised immune systems or cystic fibrosis.

Recently, a TetR-like regulator named SMLT2053 was reported in *S. maltophilia,* which shares 39% of its identity with *P. aeruginosa* PsrA. The *smlt2053* promoter has two inverted repeats that resemble the consensus motif G/CAAAC(N_2-4_)GTTTG/C of the PsrA binding site in *P. aeruginosa*. Both consensus sequences are conserved among genotypically different *S. maltophilia* strains (101).

In addition, SMLT2053 can detect and bind long-chain fatty acids such as palmitic acid (C16:0) and 13-methyltetradecanoic acid (iso-C15:0). Iso-C15:0 is the most abundant fatty acid found in *S. maltophilia* (101). In addition to its affinity for fatty acids, it has been reported that SMLT2053 can recognize and bind to its DSF molecule, cis-11-methyl-2-dodecenoic acid (101). The binding of these molecules to the SMLT2053 transcriptional factor disrupts its attachment to its promoter. It modifies its transcriptional activity, a phenomenon that has been described in PsrA of *P. aeruginosa* when it binds to long-chain fatty acids (55). As PsrA, SMLT2053 also negatively regulates fatty acid β-oxidation; here, it represses the putative fatty acid β-oxidation operon *smlt0264-smlt0268* (Fig. 3). Coves et al. (101) proposed that when SMLT2053 senses free fatty acids from the environment, it derepresses the fatty acid β-oxidation operon and degrades long-chain fatty acids. Additionally, Coves et al. (101) reported an increase in transcription of the *smlt3600-smlt3601* operon that encodes genes for iron storage behavior in a *smlt2053* mutant, indicating the negative role of Smlt2053 in iron acquisition (Fig. 3). In contrast to the *psrA* mutant in *P. aeruginosa* that does not produce biofilm, in the *S. maltophilia Δsmlt2053* strain, biofilm is overproduced, and its regulation is indirect. However, more studies are required in this regard since the formation of biofilm represents a problem for the treatment of infections by this bacterium.

The fact that SMLT2053 is capable of binding to its DSF and that its similarity to PsrA from *P. aeruginosa* raises the question of whether, in addition to sensing fatty acids, PsrA from *P. aeruginosa* could sense DSF molecules.

Although PsrA orthologs and PsrA-like regulators in different bacterial genera share the ability to sense fatty acids and modulate metabolic and virulence-associated pathways, their evolutionary relationships are not uniform. In some species, such as *Pseudomonas* and *Azotobacter*, PsrA proteins exhibit clear sequence and functional conservation, supporting a shared evolutionary origin. In contrast, regulators such as TfmR in *S. maltophilia* display low sequence identity to PsrA, despite responding to similar lipid-derived signals and controlling overlapping physiological processes.

These observations suggest that fatty acid-responsive transcriptional control has emerged through a combination of divergent evolution within the TetR family and convergent functional adaptation driven by similar ecological and metabolic pressures. In this context, PsrA should not be viewed as a universally conserved regulator across bacterial species, but rather as an example of how TetR-family regulators can be independently recruited to couple lipid metabolism with stress responses and virulence. Such functional modularity likely facilitates the integration of metabolic cues into species-specific regulatory networks shaped by distinct ecological niches and pathogenic strategies.

## OPEN QUESTIONS AND FUTURE DIRECTIONS

Since its discovery, PsrA has been extensively studied to elucidate its role in bacterial physiology. This TetR-type regulator emerged as a central transcriptional regulator that coordinates metabolism, stress responses, and virulence in T3SS-positive *P. aeruginosa* strains and related species. Despite this work, fundamental questions remain unresolved regarding how PsrA operates within the broader regulatory landscape, how its activity is controlled, and how it contributes to improving the adaptability of bacteria to their environments under fluctuating and often hostile conditions.

One major unresolved issue concerns the regulation of *psrA* expression itself. While large-scale transcriptomic analyses indicate that *psrA* is differentially expressed in mutants of several global regulators, including ExsA, LasR, GbdR, PchR, and PhoB, it remains unclear whether these effects result from direct promoter binding or indirect regulatory cascades. The absence of promoter-level mechanistic studies in *P. aeruginosa,* such as ChIP-seq, DNase I footprinting, or systematic EMSA analyses, prevents a clear understanding of how *psrA* is transcriptionally integrated into the global regulon. Although negative autoregulation of *psrA* has been demonstrated in several bacteria, including *P. putida*, *A. vinelandii,* and *L. pneumophila*, the physiological relevance, regulatory strength, and environmental conditions under which PsrA autoregulation operates in *P. aeruginosa* remain poorly defined. Equally important are the unresolved questions surrounding the environmental and metabolic signals that activate or modulate PsrA activity. Although long-chain fatty acids such as oleic acid have been shown to affect PsrA–DNA interactions, the full spectrum of ligands sensed by PsrA is unknown. It remains to be determined whether additional fatty acids, acyl-CoA intermediates, or host-derived metabolites act as signals, and how these cues are prioritized under different physiological states. Furthermore, the extent to which environmental stresses relevant to infection, such as nutrient limitation, oxidative stress, temperature shifts, or phosphate and iron availability, directly influence PsrA activity at the post-translational level is still poorly understood.

Another critical gap lies in defining the hierarchical position of PsrA within the regulatory network of *P. aeruginosa*. PsrA has been implicated in the regulation of alternative sigma factors, quorum-sensing systems, secretion systems associated with acute infection, small RNA pathways, and metabolic processes. However, how these regulatory outputs are coordinated temporally and conditionally remains unresolved. Is PsrA an upstream regulator that initiates broad transcriptional programs, or does it function primarily as an integrative node whose activity depends on upstream global regulators and environmental context? Addressing this question will require approaches that combine transcriptomics, proteomics, and metabolomics under defined environmental and infection conditions.

The role of PsrA in mediating transitions between acute and chronic infection states also remains an open question. While PsrA positively influences the T3SS and acute virulence, it also affects pathways associated with quorum sensing, biofilm formation, and metabolic adaptation. Whether PsrA actively coordinates the switch between these infection modes, or whether its regulatory influence is reshaped by other dominant regulators during chronic adaptation, has yet to be determined. Comparative studies using clinical isolates and longitudinal infection models may be particularly informative in this regard. Additionally, what is the role of PsrA in *P. aeruginosa* strains that lack the T3SS, like clades 3 and 5 of *P. aeruginosa*? Does PsrA participate in the regulation of alternative virulence mechanisms such as the ExlA two-partner secretion system or other T3SS-independent pathogenic strategies?

Despite growing interest in PsrA as a potential anti-virulence target, its contribution to antimicrobial resistance remains insufficiently characterized. Although PsrA has been linked to integron integrase expression, it is unclear whether this regulation is direct, context-dependent, or part of a broader stress adaptation program. The extent to which PsrA influences antibiotic tolerance, persistence, or resistance evolution through metabolic rewiring or stress response pathways remains an important area for future investigation.

Finally, many questions remain regarding the conservation and divergence of PsrA function across bacterial species. While core roles in stress regulation and metabolism appear conserved, the regulatory circuits and virulence-associated outputs controlled by PsrA differ substantially among genera. Moreover, structural predictions suggest conserved modular architecture, yet experimental validation of structure and function relationships is lacking. Determining how ligand binding, dimerization, and DNA recognition are structurally coupled will be essential for understanding how PsrA has evolved to fulfill species-specific regulatory roles.

Addressing these open questions will require moving beyond single-gene analyses toward integrative, systems-level studies that capture the dynamic nature of regulatory networks. Such approaches will be crucial not only for defining the precise role of PsrA in bacterial physiology and virulence but also for assessing its potential as a therapeutic target aimed at disrupting regulatory integration rather than individual virulence factors.
